# Supplementary material for: Immunosuppression causes dynamic changes in expression QTLs in psoriatic skin
Source: Nat Commun. 2023 Oct 7;14:6268. doi: 10.1038/s41467-023-41984-2 (PMC10560299; doi:10.1038/s41467-023-41984-2)
Supplement: Supplementary file 1 — Supplementary Information [file 41467_2023_41984_MOESM1_ESM.pdf]

Supplementary Information

Supplementary Figures

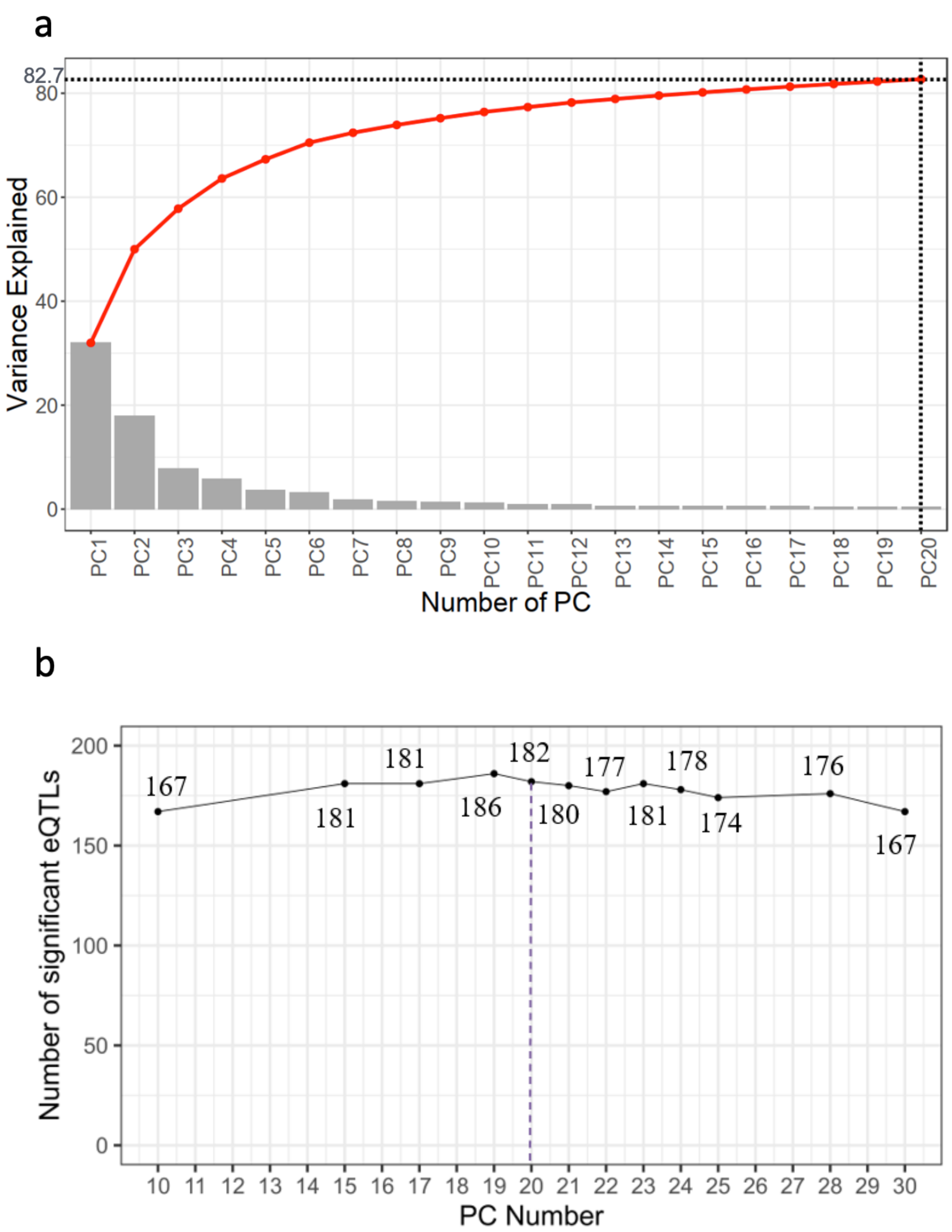

Supplementary Figure 1. eQTL mapping correcting for 20 PCs. (a) Scree plot showing the variance captured by 20 PCs. **(b)** Line plot showing the number of significant eQTLs detected using a range of PC numbers (PC10-30).

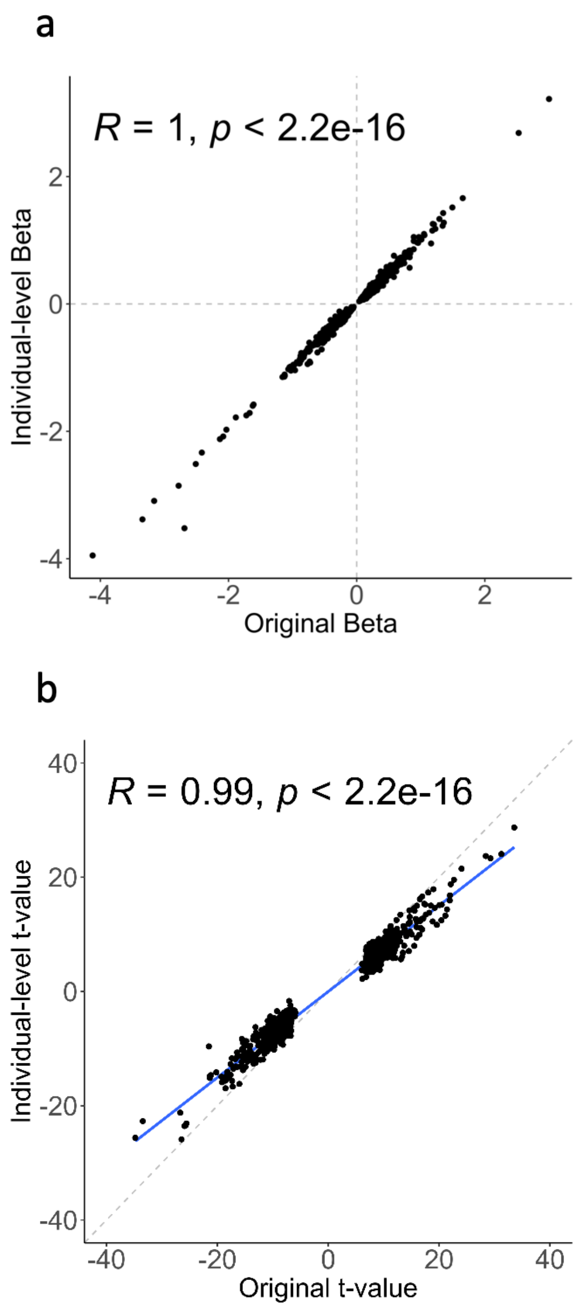

Supplementary Figure 2. First visit vs all visits. For the first visit analysis we included 140 samples (generally one lesional and one non-lesional sample) from 74 individuals. For the analysis with all visits we included 375 samples from 77 individuals. We observed 575 significant eGenes in the ($p < 6.69e-9 = 0.05/6,305,752$) for the first visit analysis versus 953 eGenes for the analysis including all visits. **(a)** Comparison of effect sizes obtained using firstvisit samples vs. all visit samples using two-sided Pearson correlation test ($p < 1e-200$) **(b)** Comparison of test statistic obtained using first visit samples vs. all visit samples using two-sided Pearson correlation test ($p < 1e-200$).

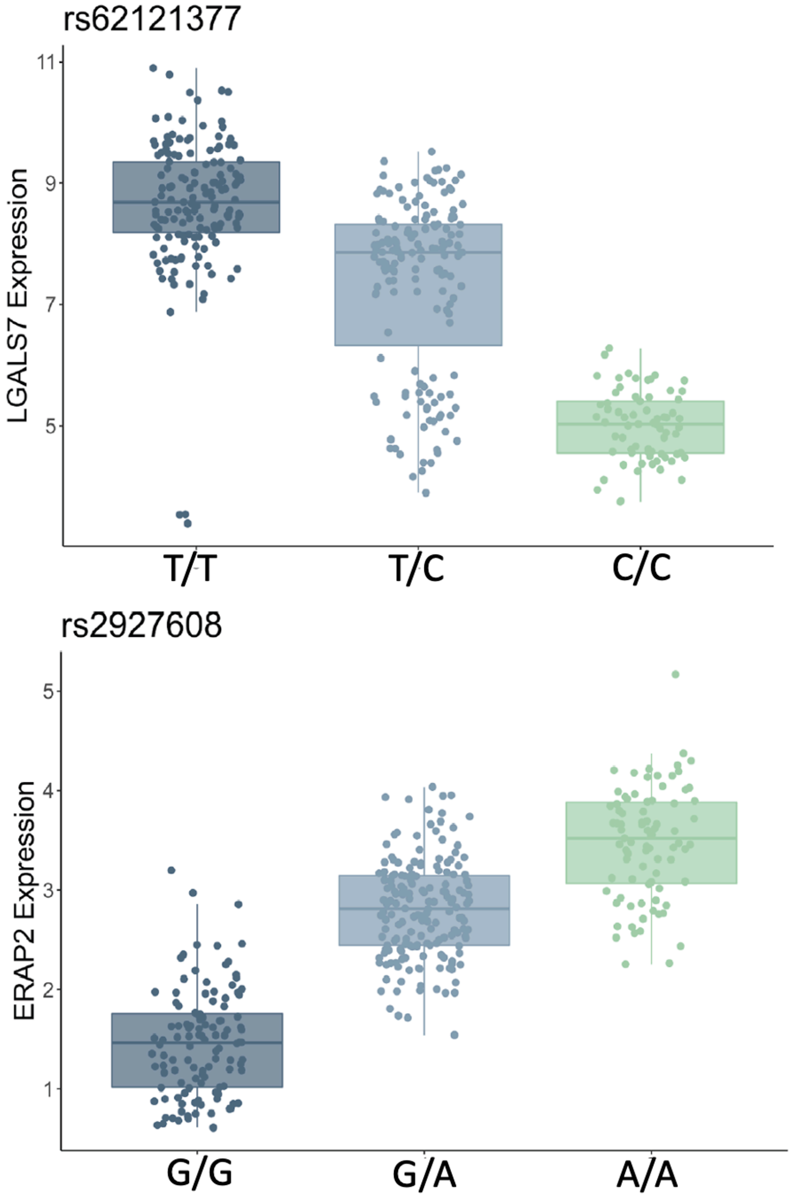

Supplementary Figure 3. Mapping eQTLs with PAUSE trial skin biopsies. Expression level of genes *LGALS7* (top) and *ERAP2* (bottom) plotted with respect to rs62121377 and rs2927608 genotypes. The middle line in the box plots show medians, and the hinges correspond to the 25th and 75th percentiles. The whiskers extend the largest and smallest value no further than 1.5 × IQR from the hinghes (n = 375 samples). For the psoriatic samples, all must be done by filtering out the smaples that don't meet the QC metrics.

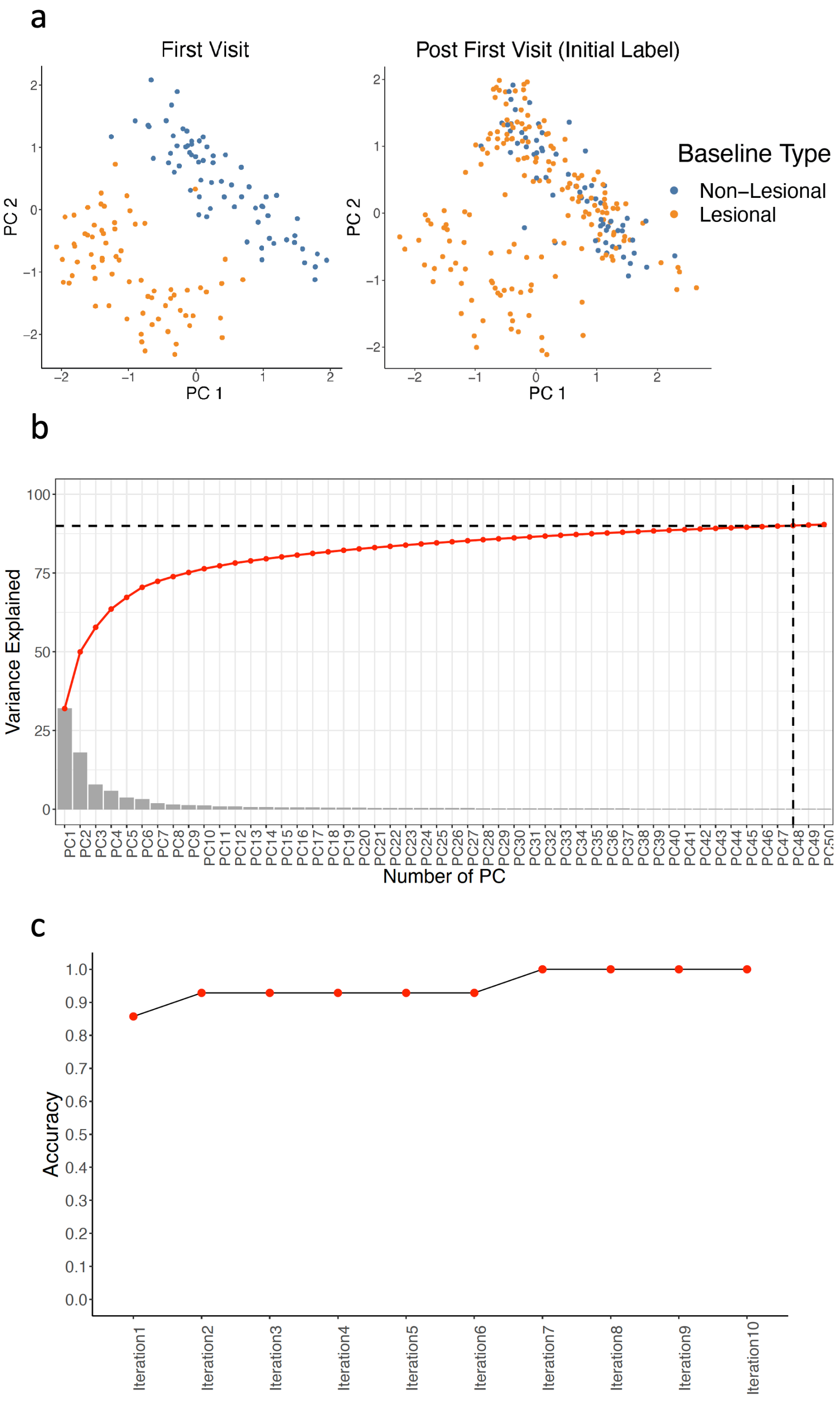

Supplementary Figure 4. Skin biopsy transcriptome in PCA space. (a) All the samples were labelled as lesional or non-lesional based on the skin status at the first visit. The first 2 RNAseq PCs illustrate the shift of transcriptional profile of lesional samples to a more non-lesional like profile as the lesion resolved over the study. **(b)** The first 48 PCs explain >90% of the variance of the transcriptional data. **(c)** Performance of LDA classifier using SPITS score to predict inflammation status in 10-fold cross-validation**.**

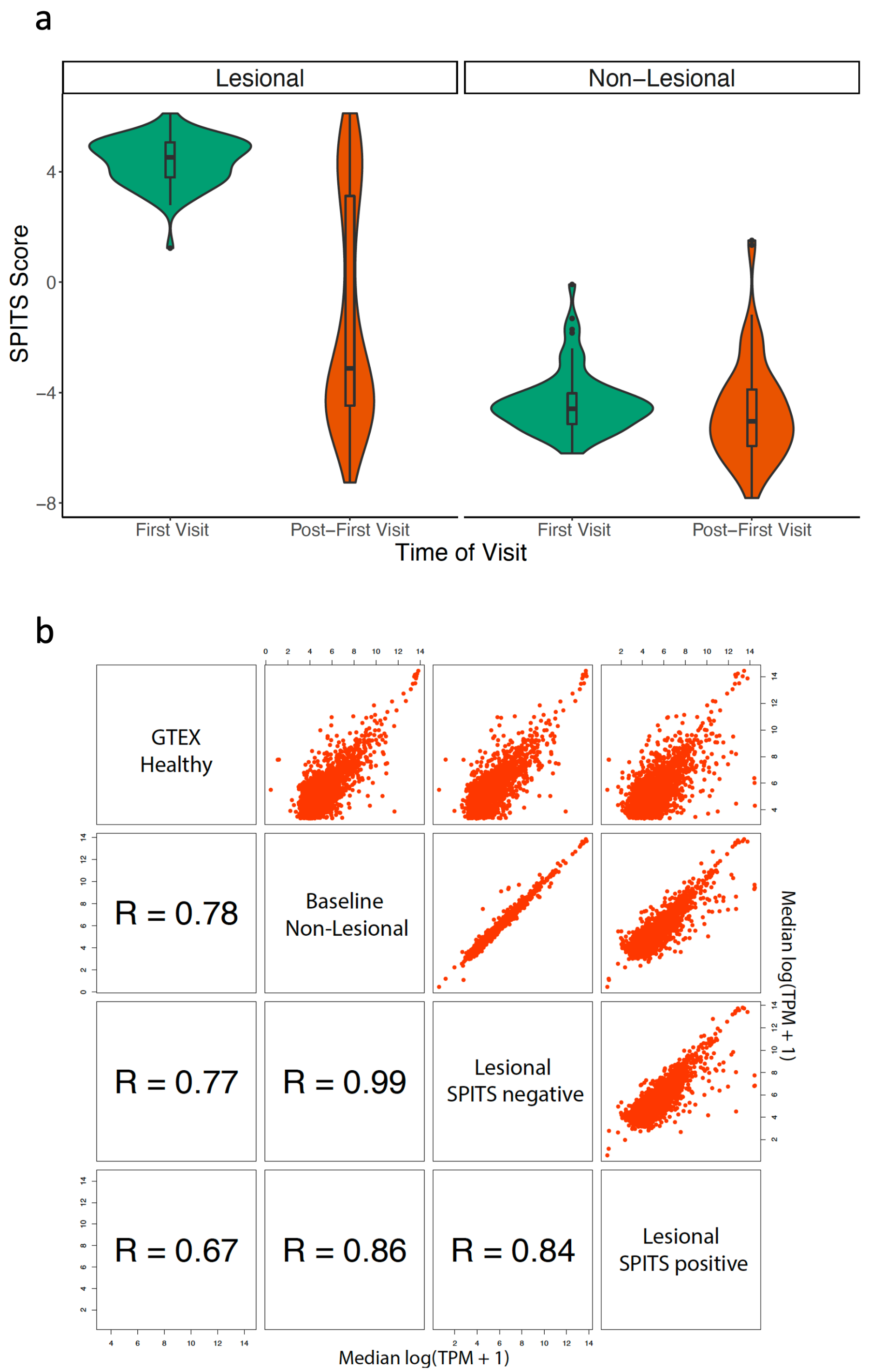

Supplementary Figure 5. SPITS distribution and correlation across samples. (a) SPITS score distribution in lesional and non-lesional samples at the first visit and after the first visit. The middle line in the box plots show medians, and the hinges correspond to the 25th and 75th percentiles. The whiskers extend the largest and smallest value no further than 1.5 × IQR from the hinghes (n = 375 samples). **(b)** Scatterplot matrix of gene expression from healthy samples, baseline non-lesional samples, SPITS negative lesional samples and SPITS positive lesional samples. Healthy skin samples from GTEx consortium[1] are more correlated with baseline non-lesional and SPITS negative lesional samples comparing to SPITS positive lesional samples.

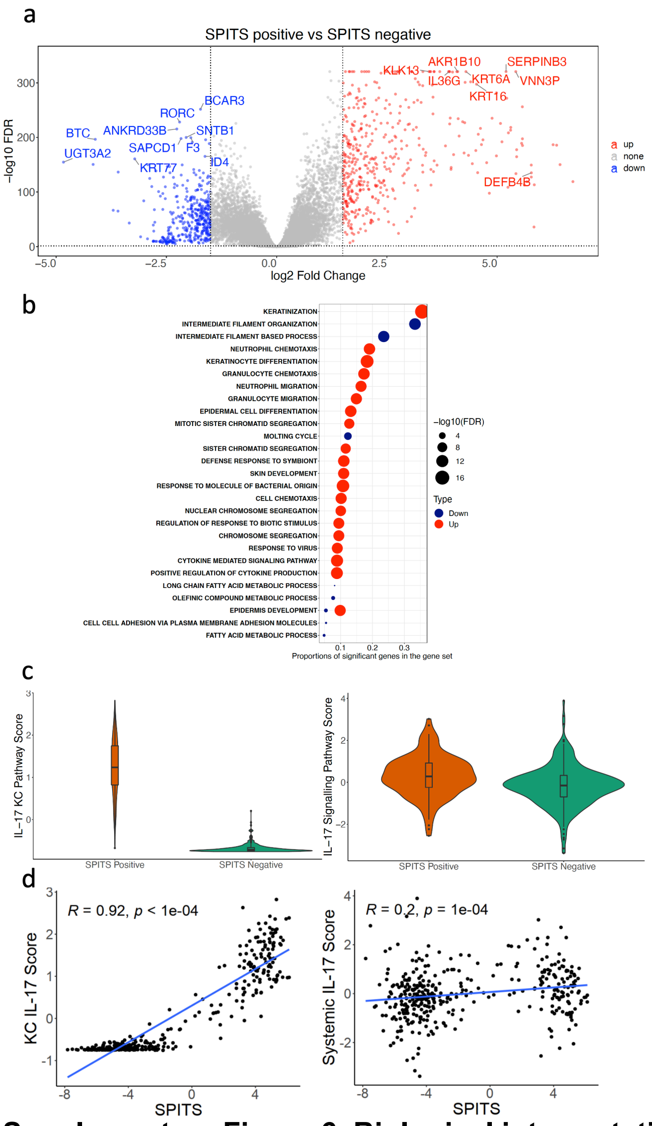

Supplementary Figure 6. Biological interpretation of SPITS. (a) Differentially expressed genes(DEGs) between SPITS positive and SPITS negative samples (N=375). Differential expression is defined by FDR < 0.05 and |logFC| > 1.5. Upregulated genes are colored in red, downregulated genes are colored in blue, and non-significant genes are colored in grey. **(b)** GO enrichement analysis of DEGs**. (c)** Violin plots showing *IL-17* pathway scores generated from induced keratinocytes[2] in SPITS positive and SPITS negative samples (left), *IL17* pathway scores generated from curated gene set[3] in SPITS positive and SPITS negative samples (right). The middle line in the box plots show medians, and the hinges correspond to the 25th and 75th percentiles. The whiskers extend the largest and smallest value no further than 1.5 × IQR from the hinghes (n = 375 samples). **(d)** Scatterplots showing *IL-17* pathway scores generated from induced keratinocytes[2] in SPITS positive and SPITS negative samples (left), *IL17* pathway scores generated from curated gene set[3] in SPITS positive and SPITS negative samples (right). The p-vaules were calculated using two-sided Pearson correlation test, the exact p-values for the left panel is p = 2.48e-157, and for the right panel is p = 1.18e-4.

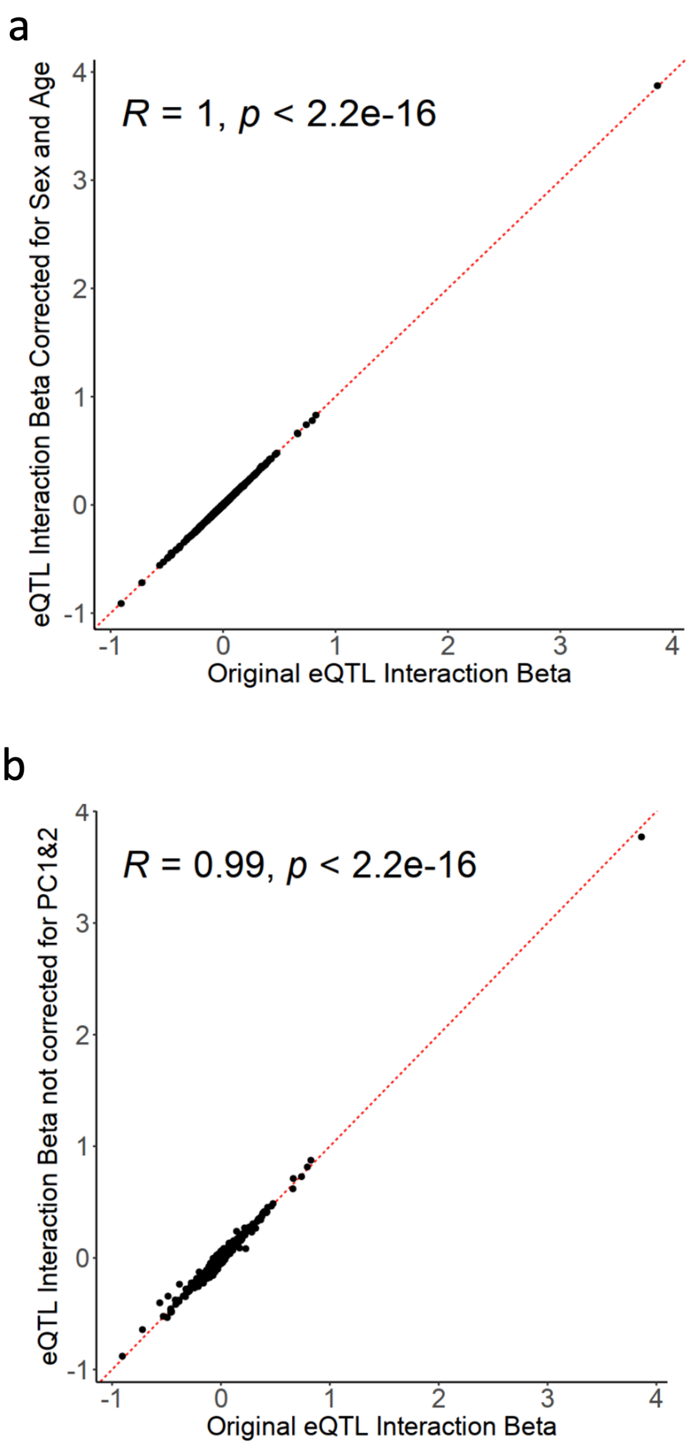

Supplementary Figure 7. SPITS interactions corrected for different covariates. (a) Scatterplot showing SPITS status-eQTL interaction beta not adjusted (x-axis) and adjusted (y-axis) for age and sex in addition to PCs. Two-sided Pearson correlation test was used ($p < 1e-200$). **(b)** Scatterplot showing SPITS status-eQTL interaction beta adjusted (x-axis) and not adjusted (y-axis) for SPITS-correlated PC1 and PC2. Two-sided Pearson correlation test was used ($p < 1e-200$).

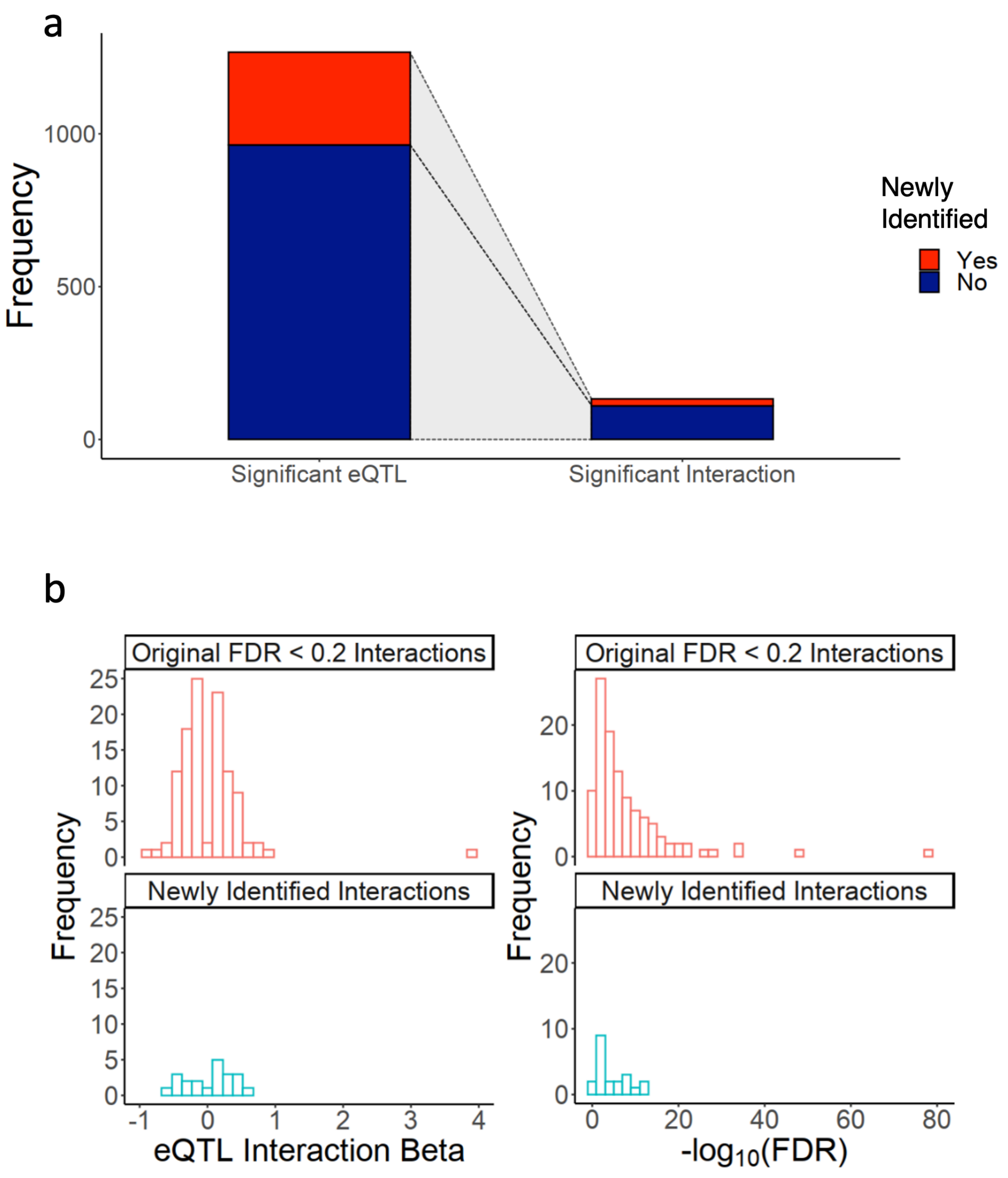

Supplementary Figure 8. Defining eQTLs using Bonferroni p-value threshold. (a) Bar plot showing the number newly identified eQTLs and eQTL interactions with a less stringent p < 1e-7 threshold in red. The number of signals detected using Bonferroni threshold is in blue. **(b)** Histograms showing interaction betas (left) and FDR distributions (right) of eQTL interactions detected by applying Bonferroni threshold (top), or p < 1e-7 (bottom).

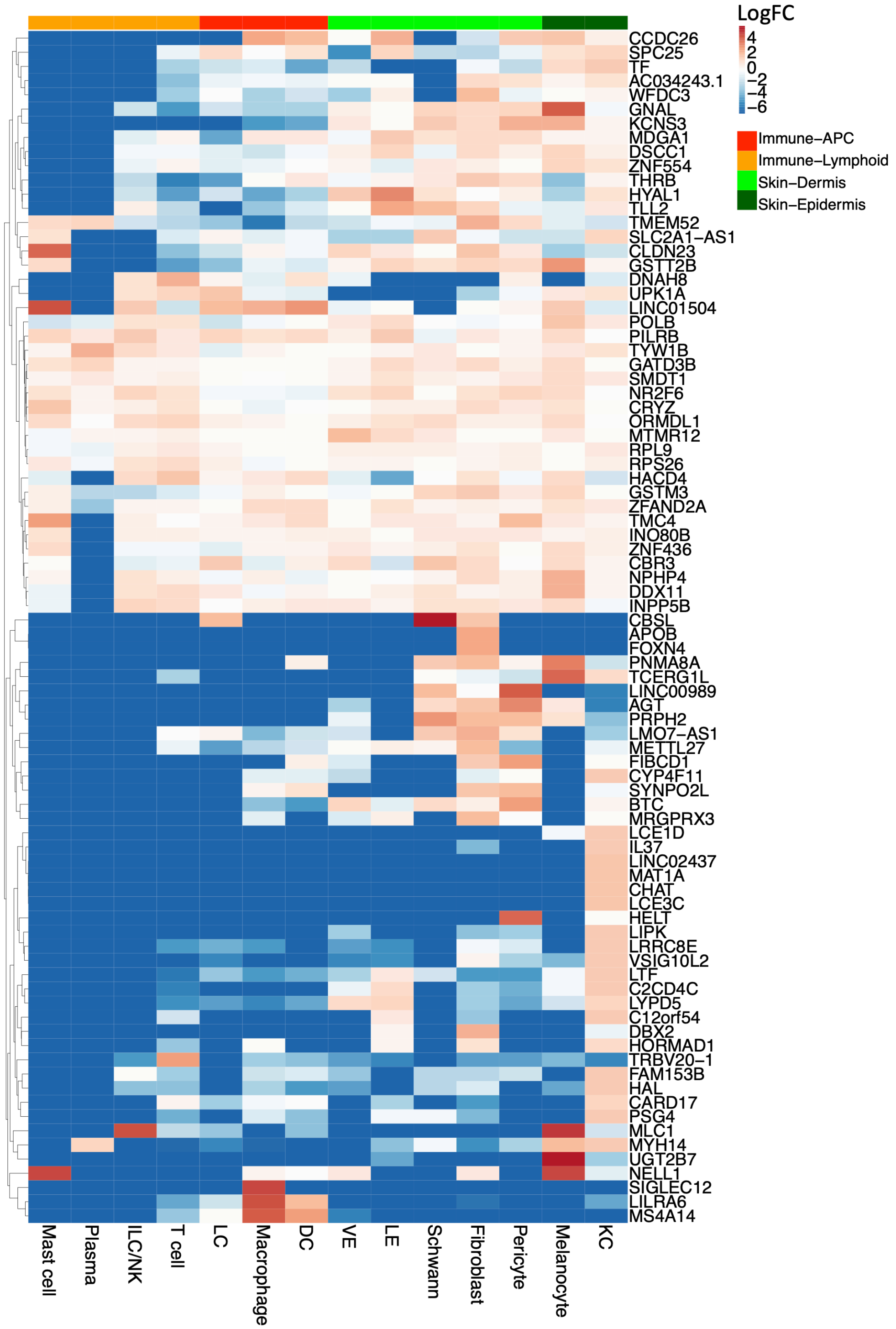

Supplementary Figure 9. Log fold change of interacting eGene expression per cell type against mean eGene expression across the 14 cell types. The cell types are classified into 4 categories: immune-antigen presenting cells (APC), immune-lymphoid, skin-epidermis and skin-dermis.

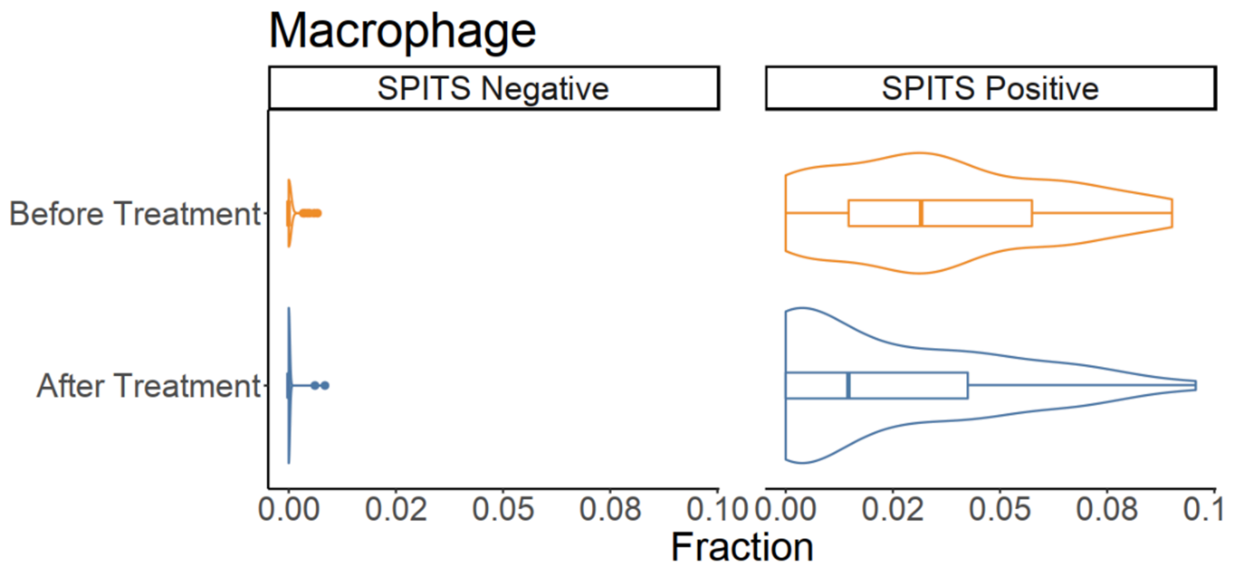

Supplementary Figure 10. The shift of inferred macrophage fraction in the sample before and after treatment. The middle line in the box plots show medians, and the hinges correspond to the 25th and 75th percentiles. The whiskers extend the largest and smallest value no further than 1.5 × IQR from the hinghes (n = 375 samples).

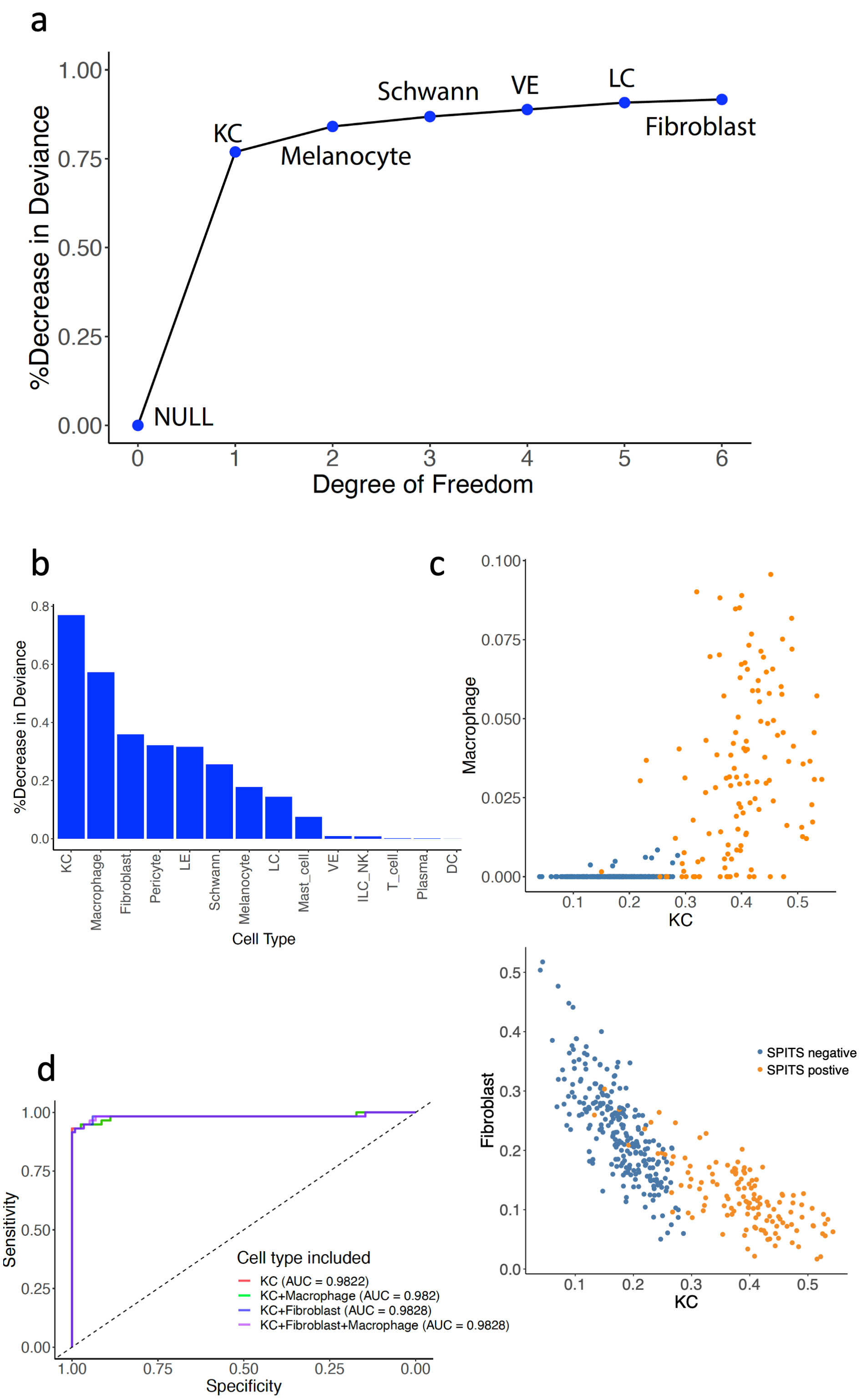

Supplementary Figure 11. Cell type composition predicts SPITS. (a) %Decrease in deviance for the selected logistic model. KC, keratinocyte; VE, vascular endothelium; LC, Langerhans cells. **(b)** Decrease in deviance for univariate models. **(c)** Keratinocyte (KC), macrophage and fibroblast fractions separate inferred inflammation status. **(d)** ROC curves evaluating LDA classifiers using different cell types as predictors.

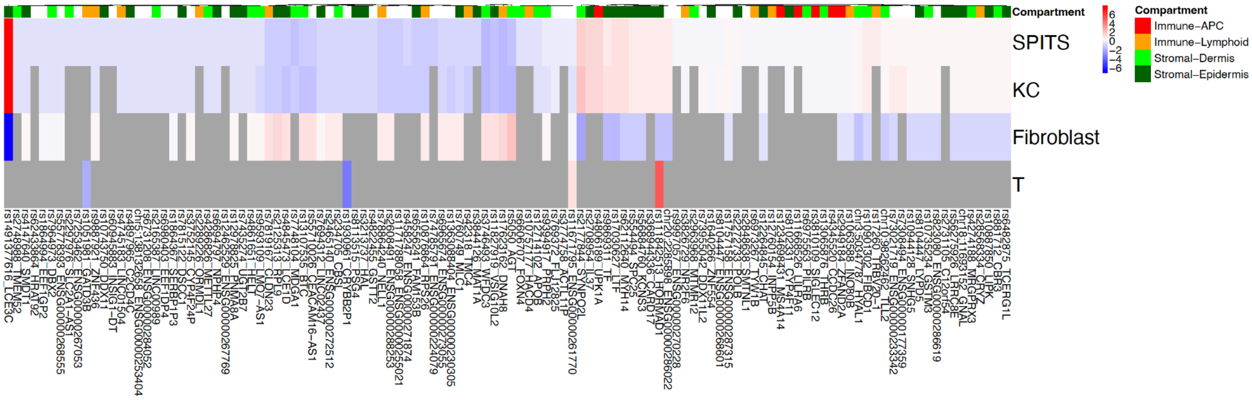

Supplementary Figure 12. Heatmap of all SPITS eQTL interactions, in comparison with KC, fibroblast, and T cell interactions. The values are the absolute value of interaction coefficient z-scores. The dampeners are colored in blue, the magnifiers colored in red, and non-significant interactions are in grey. Tissue specificity of the interactions are annotated using the same eGene tissue-specificity identified with single-cell data. No tissue specificity labeled if the eGene was not present in the single-cell data.

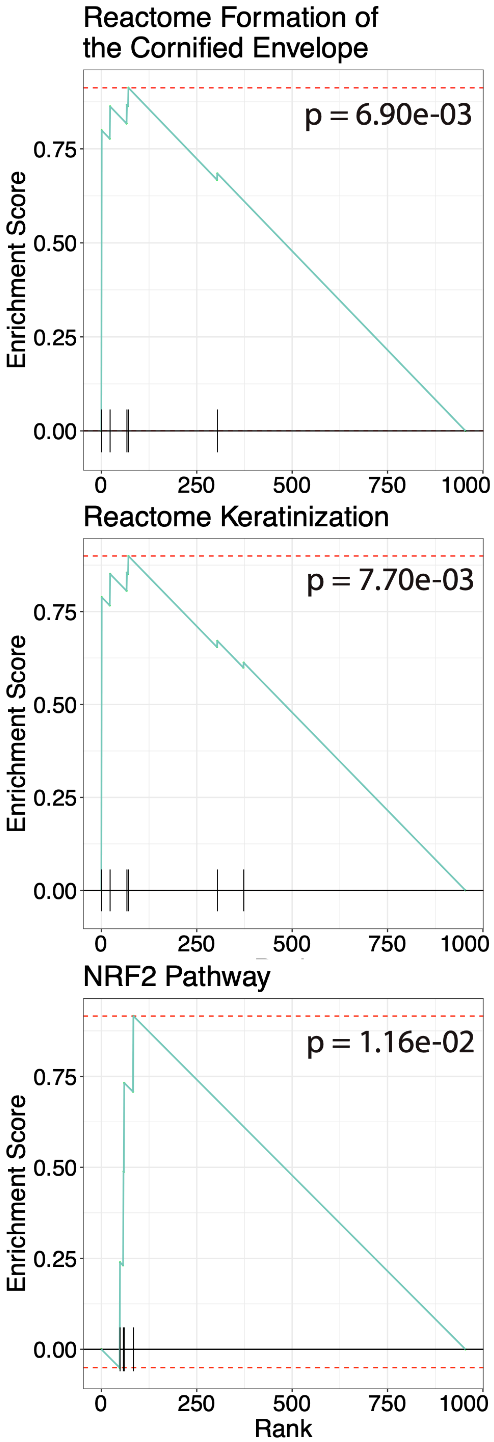

Supplementary Figure 13. Gene set enrichment analysis of SPITS-interaction eGenes. The genes are ranked by absolute value of interaction beta, and p-values are obtained from 10000 permutations.

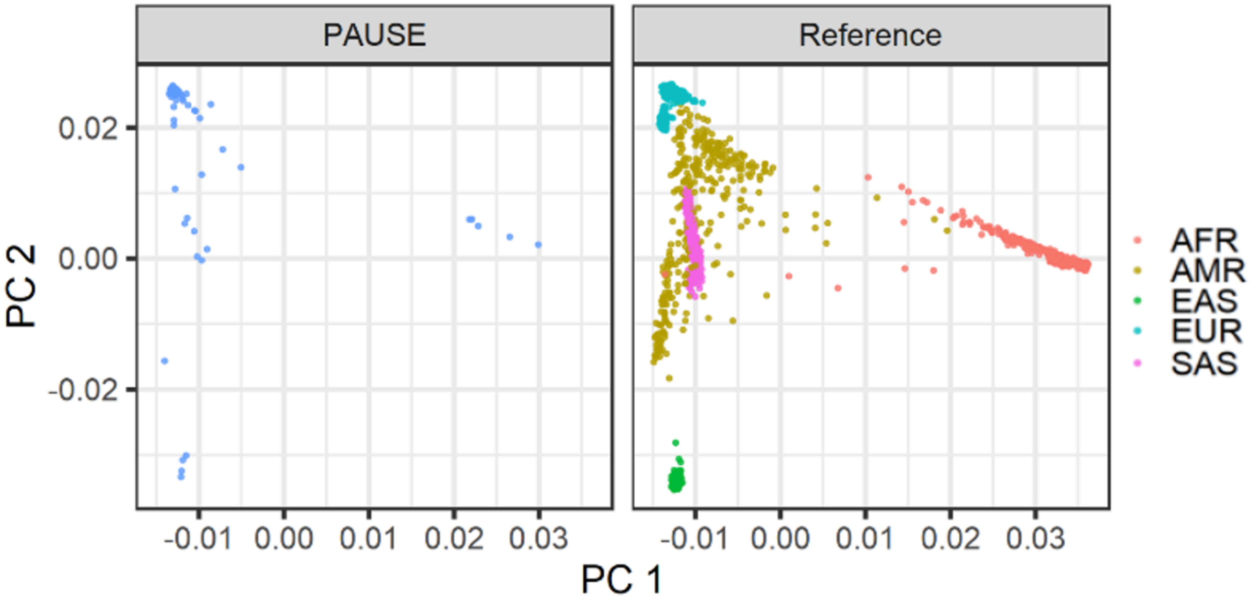

Supplementary Figure 14. Ancestry inference of PAUSE samples from 1000 Genomes and HapMap data.

	Number (%) of Participants	
**Characteristic**	**Total (n = 108)**	**Included for eQTL analysis (n = 77)**
Age, mean (SD), y	46.1 (12.08)	47.2 (11.8)
Sex		
Male	73 (67.6)	51 (66.2)
Female	35 (32.4)	26 (33.8)
Race		
White or Caucasian	95 (88.0)	64 (83.1)
Black or African American	6 (5.6)	6 (7.8)
Asian	4 (3.7)	4 (5.2)
Other	1 (0.9)	2 (2.6)
Multiple Races	2 (1.9)	1 (1.3)
Ethnicity		
Hispanic or Latino	12 (11.1)	6 (7.8)
Not Hispanic or Latino	96 (88.9)	71 (92.2)
Weight, mean (SD), kg	97.5 (21.48)	96.7 (21.68)
Height, mean (SD), cm	171.9 (9.87)	171.5 (10.19)
BMI, mean (SD), kg/m^2	33.0 (6.88)	32.9 (7.21)
Week 0 PASI		
Number	108	74
Mean (SD)	19.9 (8.06)	19.9 (8.50)
12-20	71 (65.7)	49 (67.6)
>20	37 (34.3)	24 (32.4)
Week 12 PASI		
Number	103	70
Mean (SD)	3.1 (4.27)	2.1 (1.99)

Supplementary Table 1. Population characteristics of the cohort from the PAUSE trial (n = 108) and the individuals included for eQTL analysis (n = 77).
