# Supplementary material for: Immunosuppression causes dynamic changes in expression QTLs in psoriatic skin
Source: Nat Commun. 2023 Oct 7;14:6268. doi: 10.1038/s41467-023-41984-2 (PMC10560299; doi:10.1038/s41467-023-41984-2)
Supplement: Supplementary file 3 — Description of Additional Supplementary Files [file 41467_2023_41984_MOESM3_ESM.pdf]

Description of Additional Supplementary Files

File Name: Supplementary Data 1
Description: Counts of skin biopsies from each donor conditioned on lesional status and treatment status.

File Name: Supplementary Data 2
Description: 953 significant cis-eQTLs detected from the 375 skin biopsies using linear mixed models, passing the Bonferroni threshold ($p < 6.69e-9$). The table is sorted by decreasing p-value.

File Name: Supplementary Data 3
Description: GTEx (GTEx Consortium, 2015) and PAUSE skin eQTL summary statistics merged upon same eGenes. Consistency of eQTLs coefficient signs between the two studies is recorded.

File Name: Supplementary Data 4
Description: PAUSE eQTLs colocalized with various skin traits (PP.H4 > 0.75) calculated from summary statistics obtained from GWAS Catalog (https://www.ebi.ac.uk/gwas/). The first sheet records colocalizations between PAUSE eQTLs and psoriasis GWAS (Tsoi et al., 2012, GCST005527), the second sheet records colocalizations between PAUSE eQTLs and eczema GWAS (Grosche et al., 2021, GCST90044763), and the third sheet records colocalizations between PAUSE eQTLs and scleroderma GWAS (Lopez-Isac et al., 2019, GCST009131).

File Name: Supplementary Data 5
Description: Differentially expressed genes ($FDR < 0.05$, $|logFC| > 1.5$) between SPITS positive and SPITS negative skin samples ($N = 375$).

File Name: Supplementary Data 6
Description: Summary statistics of the 953 detected cis-eQTLs interacting with SPITS (sheet 1), treatment group (sheet 2), PASI score (sheet 3), IL-17 score (sheet 4), keratinocyte proportions (sheet 5), T cell proportions (sheet 6), and fibroblast proportions (sheet 7).
