# Supplementary material for: Immunosuppression causes dynamic changes in expression QTLs in psoriatic skin
Source: Nat Commun. 2023 Oct 7;14:6268. doi: 10.1038/s41467-023-41984-2 (PMC10560299; doi:10.1038/s41467-023-41984-2)
Supplement: Supplementary file 10 — Reporting Summary [file 41467_2023_41984_MOESM10_ESM.pdf]

nature portfolio

Corresponding author(s): Soumya Raychaudhuri

Last updated by author(s): Jul 7, 2023

Reporting Summary

Nature Portfolio wishes to improve the reproducibility of the work that we publish. This form provides structure for consistency and transparency in reporting. For further information on Nature Portfolio policies, see our Editorial Policies and the Editorial Policy Checklist.

Statistics

For all statistical analyses, confirm that the following items are present in the figure legend, table legend, main text, or Methods section.

n/a	Confirmed	
☐	☒	The exact sample size (*n*) for each experimental group/condition, given as a discrete number and unit of measurement
☐	☒	A statement on whether measurements were taken from distinct samples or whether the same sample was measured repeatedly
☐	☒	The statistical test(s) used AND whether they are one- or two-sided<br>*Only common tests should be described solely by name; describe more complex techniques in the Methods section.*
☐	☒	A description of all covariates tested
☐	☒	A description of any assumptions or corrections, such as tests of normality and adjustment for multiple comparisons
☐	☒	A full description of the statistical parameters including central tendency (e.g. means) or other basic estimates (e.g. regression coefficient) AND variation (e.g. standard deviation) or associated estimates of uncertainty (e.g. confidence intervals)
☐	☒	For null hypothesis testing, the test statistic (e.g. *F*, *t*, *r*) with confidence intervals, effect sizes, degrees of freedom and *P* value noted<br>*Give P values as exact values whenever suitable.*
☐	☒	For Bayesian analysis, information on the choice of priors and Markov chain Monte Carlo settings
☐	☒	For hierarchical and complex designs, identification of the appropriate level for tests and full reporting of outcomes
☐	☒	Estimates of effect sizes (e.g. Cohen's *d*, Pearson's *r*), indicating how they were calculated

Our web collection on statistics for biologists contains articles on many of the points above.

Software and code

Policy information about availability of computer code

Data collection	No software was used for data collection
Data analysis	PLINK (v.1.90), SHAPEIT (v.2.727), minimac3 (v.2.0.1), HOMER (v.4.11), CIBERSORTx (v.1.0), R (v.4.3.0). Subsequent analysis was done by custom scripts and code, made available via github: https://github.com/immunogenomics/PAUSE_eQTL

For manuscripts utilizing custom algorithms or software that are central to the research but not yet described in published literature, software must be made available to editors and reviewers. We strongly encourage code deposition in a community repository (e.g. GitHub). See the Nature Portfolio guidelines for submitting code & software for further information.

Data

Policy information about availability of data

All manuscripts must include a data availability statement. This statement should provide the following information, where applicable:

- Accession codes, unique identifiers, or web links for publicly available datasets
- A description of any restrictions on data availability
- For clinical datasets or third party data, please ensure that the statement adheres to our policy

The raw gene expression and sequencing data is deposited in dbGap (accession code: phs003395). The data will be available upon request, by submitting a letter of intent to the corresponding author. The clinical data of PAUSE trial is available on the ITN TrialShare website (www.itntrialshare.org). GTEx eQTL(https://storage.googleapis.com/gtex_analysis_v7/single_tissue_eqtl_data/all_snp_gene_associations/Skin_Not_Sun_Exposed_Suprapubic.allpairs.txt.gz) and median TPM

data(https://storage.googleapis.com/gtex_analysis_v8/rna_seq_data/GTEx_Analysis_2017-06-05_v8_RNASeQCv1.1.9_gene_median_tpm.gct.gz) were obtained from GTEx portal (gtexportal.org). The skin scRNAseq data was obtained from ArrayExpress (https://www.ebi.ac.uk/arrayexpress/experiments/E-MTAB-8142). GWAS summary statistics were downloaded from GWAS catalog (www.ebi.ac.uk/gwas), including psoriasis (GCST005527), systemic scleroderma (GCST009131) and eczema (GCST90044763).

Research involving human participants, their data, or biological material

Policy information about studies with human participants or human data. See also policy information about sex, gender (identity/presentation), and sexual orientation and race, ethnicity and racism.

Reporting on sex and gender	See original clinical trial: https://www.ncbi.nlm.nih.gov/pmc/articles/PMC8515260/ Recruitment was not restricted by gender or sex
Reporting on race, ethnicity, or other socially relevant groupings	See original clinical trial: https://www.ncbi.nlm.nih.gov/pmc/articles/PMC8515260/
Population characteristics	Data were obtained from individuals with psoriasis from PAUSE trial. The mean age is 47.2 (SD = 11.8). The population is composed of 66.2% male and 33.8% female. More details could be find in Supplementary Table 1.
Recruitment	The participants were enrolled from March 2014 to April 2016 at 10 investigational sites in the United States and Canada. All participants provided written informed consent. Race and ethnicity were self-reported or investigator observed. This unlikely impacts our results because the analysis didn't rely on self-reported race/ethnicity information.
Ethics oversight	All participants provided written informed consent. The trial was conducted in compliance with the Declaration of Helenski and was approved by the institutional review boards at all of the investigational sites (US: Dermatology Research Associates, Los Angeles, California; Northwestern University , Chicago, Illinois; Tulane University School of Medicine, New Orleans ,Louisiana; University of Michigan, Ann Arbor; The Rockefeller University, New York, New York; Wake Forest University, Winston-Salem, North Carolina; Case Western University, Cleveland, Ohio; and University of Utah, Salt Lake City; Canada: Kirk Barber Research, Calgary, Alberta, and Innovaderm Research Inc, Montreal, Quebec)

Note that full information on the approval of the study protocol must also be provided in the manuscript.

Field-specific reporting

Please select the one below that is the best fit for your research. If you are not sure, read the appropriate sections before making your selection.

☒ Life sciences ☐ Behavioural & social sciences ☐ Ecological, evolutionary & environmental sciences

For a reference copy of the document with all sections, see nature.com/documents/nr-reporting-summary-flat.pdf

Life sciences study design

All studies must disclose on these points even when the disclosure is negative.

Sample size	Sample size used for this study is 375 samples from 77 individuals. The size were chosen as they were the available samples that meet QC criteria.
Data exclusions	5 individuals were removed due to high missingness (> 10%) and filtered SNPs with call rate < 0.99, MAF < 0.05, or Hardy-Weinberg Equilibrium (HWE) p < 1e-6. An additional 19 individuals were removed due to a lack of or low-quality RNA-seq data, leaving 77 patients for eQTL analyses.
Replication	By comparing the significant eQTLs identified in this study to GTEx, we found the replication rate to be 98.5%.
Randomization	Randomized to treatment
Blinding	Double blinded

Reporting for specific materials, systems and methods

We require information from authors about some types of materials, experimental systems and methods used in many studies. Here, indicate whether each material, system or method listed is relevant to your study. If you are not sure if a list item applies to your research, read the appropriate section before selecting a response.

Materials & experimental systems

n/a	Involved in the study
☒	☐ Antibodies
☒	☐ Eukaryotic cell lines
☒	☐ Palaeontology and archaeology
☒	☐ Animals and other organisms
☐	☒ Clinical data
☒	☐ Dual use research of concern
☒	☐ Plants

Methods

n/a	Involved in the study
☒	☐ ChIP-seq
☒	☐ Flow cytometry
☒	☐ MRI-based neuroimaging

Clinical data

Policy information about clinical studies

All manuscripts should comply with the ICMJE guidelines for publication of clinical research and a completed CONSORT checklist must be included with all submissions.

Clinical trial registration	ClinicalTrials.gov Identifier: NCT01999868
Study protocol	See https://www.ncbi.nlm.nih.gov/pmc/articles/PMC8515260/
Data collection	Eligible participants were 18 to 65 years of age with a diagnosis of moderate to severe plaque psoriasis. Race and ethnicity of participants were self-reported or investigator observed. The PASI was assessed every 4 weeks until the final study visit. Skin biopsies were obtained from an active lesion and a nonlesional area at week 0 and stored in RNAlater (Ambion Inc) at −70 to −80 °C. The same lesion was resampled at weeks 12, 24 (optional), and 40 and at the final study visit, and RNA was isolated using RNeasy kits (Qiagen). Participants were genotyped across 1,748,250 variants using the Infinium Multi-Ethnic Global BeadChip from Illumina.
Outcomes	The primary endpoint was the proportion of participants with psoriasis relapse (loss of 50% of the initial PASI improvement) between weeks 12 and 88. Secondary endpoints included time to psoriasis relapse, proportion of participants with psoriasis relapse between weeks 12 and 40, and adverse events.
